# A Coding Variant in *TMC8* (EVER2) Is Associated with High Risk HPV Infection and Head and Neck Cancer Risk

**DOI:** 10.1371/journal.pone.0123716

**Published:** 2015-04-08

**Authors:** Caihua Liang, Karl T. Kelsey, Michael D. McClean, Brock C. Christensen, Carmen J. Marsit, Margaret R. Karagas, Tim Waterboer, Michael Pawlita, Heather H. Nelson

**Affiliations:** 1 Department of Epidemiology, Division of Biology and Medicine, Brown University, Providence, RI, United States of America; 2 Department of Pathology and Laboratory Medicine, Division of Biology and Medicine, Brown University, Providence, RI, United States of America; 3 Department of Environmental Health, School of Public Health, Boston University, Boston, MA, United States of America; 4 Department of Epidemiology, Dartmouth Medical School, Lebanon, NH, United States of America; 5 Norris Cotton Cancer Center, Dartmouth Medical School, Lebanon, NH, United States of America; 6 Department of Toxicology, Dartmouth Medical School, Lebanon, NH, United States of America; 7 Infection and Cancer Program, German Cancer Research Center (Deutsches Krebsforschungszentrum), Heidelberg, Germany; 8 Masonic Cancer Center, University of Minnesota, Minneapolis, MN, United States of America; 9 Division of Epidemiology and Community Health, University of Minnesota, Minneapolis, MN, United States of America; Virginia Commonwealth University, UNITED STATES

## Abstract

HPV infection is a causal agent in many epithelial cancers, yet our understanding of genetic susceptibility to HPV infection and resultant cancer risk is limited. Epidermodysplasia Verruciformis is a rare condition of extreme susceptibility to cutaneous HPV infection primarily attributable to mutations in *TMC6* and *TMC8*. Genetic variation in the *TMC6/TMC8* region has been linked to beta-type HPV infection and squamous cell carcinoma of the skin, cervical cancer, HPV persistence and progression to cervical cancer. Here, we have tested the hypothesis that the common *TMC8* SNP rs7208422 is associated with high-risk HPV infection and risk of head and neck squamous cell carcinoma (HNSCC). Seropositivity to the HPV L1 protein (HPV16, 18, 11, 31, 33, 35, 45, 52, 58) was measured in 514 cases and 452 population-based controls. Genotype was significantly associated with seropositivity to HPV18 L1 (OR _TT vs AA_ = 0.48, 95% CI = 0.22–0.99) and borderline significantly associated with HPV16 L1 (OR _TT vs AA_ = 0.58, 95% CI = 0.22–1.17). There was a consistent inverse association between *TMC8* genotype and infection with other HPV types, including statistically significant associations for HPV31 and HPV52. Consistent with these results, the variant T genotype was associated with a reduced risk of HNSCC (OR_AT_: 0.63, 95% CI 0.45–0.89, OR_TT_: 0.54, 95% CI 0.36–0.81), even among subjects seronegative for all HPV types (OR_AT_: 0.71, 95% CI 0.45–1.11, OR_TT_: 0.54, 95% CI 0.31–0.93). Our data indicate that common genetic variation in *TMC8* is associated with high-risk HPV infection and HNSCC etiology.

## Introduction

Null mutations in *TMC6* and *TMC8* confer extreme susceptibility to infection with cutaneous (beta-type) HPV. These genes encode EVER1 and EVER2 which form a heterodimer that interacts with the zinc transporter molecule ZnT-1 to control zinc levels within the cell [1]. The rare syndrome Epidermodysplasia Verrucciformis (EV) is associated with mutation in either of these genes and is characterized by large numbers of flat-like lesions infected with beta-HPVs, mainly HPV5 and HPV8 virus. Previously, we observed that genetic variation in *TMC8* was associated with seropositivity to cutaneous (beta) HPV and increased risk of squamous cell skin cancer (SCC) [2]. The observations from EV and skin cancer patients suggest that the *TMC6* and *TMC8* genes could be important factors for host protection from papillomaviruses. While the complex of EVER proteins is known to regulate zinc homeostasis in keratinocytes, it has recently been demonstrated that the *TMC6/8* genes are also expressed at high levels in T-lymphocytes [3,4]. Therefore, genetic variation in the *TMC6/8* genes may impact HPV infection and cancer risk either directly by modulating HPV infection in epithelial cells, or indirectly through immune surveillance.

Although common genetic variation in *TMC8* has been associated with beta-HPV infection, it is also possible that *TMC8* genetic variation contributes to high-risk HPV infection and attendant cancer risk (cervical, other anogenital, and head and neck malignancies). In fact, work by Wang et al [5] using dense genotyping methodology identified a rare SNP in the *TMC6*/*TMC8* gene region as being associated with HPV16 persistence. A more recent study by Castro et al [6], demonstrated that two common SNPs in the TMC6/TMC8 gene were associated with cervical cancer (in the absence of other HPV measurements). Whether common variation in this gene region confers susceptibility to high-risk HPV infection remains incompletely understood. In addition, the role of this gene in other HPV-related cancers, such as oropharyngeal cancer (rev in [7]), has not been tested. Here, we sought to determine whether the common SNP rs7208422 in *TMC8* is associated with seropositivity to high-risk HPV types and risk of HNSCC.

## Materials and Methods

### Ethics Statement

Study protocols and materials for recruitment of cases and controls were approved by the Institutional Review Boards at the nine medical facilities and Brown University. Written informed consent was obtained from all study subjects.

### Study subjects

This population-based case-control study was conducted in Greater Boston, MA between December 1999 and December 2003 and has been previously described [8]. Cases were HNSCC patients identified from head and neck clinics and departments of otolaryngology or radiation oncology at nine medical facilities in the study area during the study period. Eligible cases were residents in the study area aged 18 years or older and with a pathologically confirmed diagnosis of HNSCC no more than 6 months before the time of patient contact. Patients with carcinoma *in situ*, lip, salivary gland, or nasooropharyngeal cancer or recurrent HNSCC were excluded. The cancer registry was queried to insure that all eligible cases in the area were identified. HNSCC cases included diagnosis codes 141–146, 148, 149, and 161 according to International Classification of Disease, Ninth Revision (ICD-9). Cases were further classified by site into oral (ICD-9 codes 143–145), oropharygeal (ICD-9 codes 146, 148, 149), and laryngeal (ICD-9 code 161) disease. Controls were frequency-matched to cases on age (± 3 years), gender, and town of residence identified from Massachusetts town books through systematic random selection. The town book includes all the residents more than 17 years of age listed by street address and precincts. A potential control of the same gender and age as the case was sought from this list, alternating the search direction through the book starting at the case address.

### Detection of antibodies by multiplex serology

Venous blood samples were obtained from cases and controls. Serum was separated within 12–24 hr of blood drawing and samples were stored at -80°C. To detect antibodies against HPV, a glutathione S-transferase (GST) capture enzyme-linked immunosorbent assay (ELISA) was used, in combination with fluorescent bead technology, as described in detail previously [9,10,11,12]. Briefly, full-length viral proteins fused with an N-terminal GST domain were expressed in *E*. *coli* bacteria. Glutathione cross-linked to casein was coupled to fluorescence-labeled polystyrene beads (SeroMAP Microspheres, Luminex Corp., Austin, TX) and GST fusion proteins were *in situ* affinity-purified on the beads directly in a one-step procedure. Each fusion protein was bound to a spectrally distinct bead set. Fusion protein-loaded bead sets were mixed and incubated with human sera. Antibodies bound to the beads via the viral antigens were detected with biotinylated goat-anti-human IgG (H+L) secondary antibody and streptavidin-R-phycoerythrin. A Luminex 100 analyzer (Luminex Corp., Austin, TX) was used to identify the internal color of the individual beads and to quantify their reporter fluorescence (expressed as median fluorescence intensity (MFI) of at least 100 beads per set per serum).

MFI values were dichotomized as antibody positive or negative. A standardized cut-off for HPV16, 18, 31, 33, 35, 45, 52 and 58 L1 antibodies was defined earlier [13] and applied here. Seropositivity cut-offs for HPV16 early proteins were determined using a similar algorithm for serum samples of 117 female, HPV DNA negative, self-reported virgins from a cross-sectional study among Korean students [13]. The mean plus 5 standard deviations was calculated, and the resulting single cut-off was doubled to stringently separate seropositive and-negative reactions (HPV 16 E6, 484 MFI; HPV 16 E7, 1096 MFI) [14].

### TMC8 genotype detection

We collected data on a (A > T) coding SNP in exon 8, codon 306 of the *TMC8* gene (rs7208422), resulting in a change from asparagine to isoleucine. DNA was extracted from buffy coat using Qiagen genomic DNA extraction kits. Genotyping of the *TMC8* polymorphism was done using the Taqman allelic discrimination technique (Applied Biosystems, Foster City, CA), as previously published [2]. Known genotype controls were included for each genotyping assay. For quality assessment, every tenth sample was an embedded duplicate, and there was 100% concordance among QA genotypes.

### Statistical analysis

Data analysis was restricted to Caucasian subjects with both *TMC8* genotype and HPV serology data. To estimate the associations between *TMC8* genotype and HNSCC, we calculated odds ratios (ORs) and 95% confidence intervals (CIs) from unconditional logistic regression models. Models included the study matching factors gender and age, however the third matching parameter, town of residence, was not included in these unconditional regression models given the number of towns represented. Risk models were also adjusted for smoking (pack-years) and drinking (average drinks per week). We additionally performed the analysis stratified by tumor site as well as by HPV16 serology status. To examine the relationship between *TMC8* genotype and HPV serologic markers, we used logistic regression with positive serology as the response variable and *TMC8* genotype as an independent variable. All statistical analyzes were performed using SAS9.1, and all statistical tests were two-sided.

## Results

Among the 775 subjects, 361 were cases with HNSCC (148 oral cavity cancers, 148 oropharyngeal cancers, 65 laryngeal cancers) and 414 were control subjects (Table 1). There were no significant differences in the distributions of age and gender between cases and controls (Table 1). Most subjects were males (74%). Compared to controls, HNSCC cases were more likely to smoke (P<0.001) and drink heavily (P<0.001), and had a higher prevalence of seropositivity to HPV16 (P<0.0001), HPV18 (p<0.04), HPV11 (p<0.003), HPV31 (P<0.01), HPV35 (p<0.001), HPV52 (p<0.0003) and HPV58 (p<0.003) (Table 1).

**Table 1:** Demographic characteristics of cases and controls.

Characteristic	Cases (n = 361)	Controls (n = 414)	P
Age (years)	59.7 ± 11.5	61.0 ± 11.6	0.11
**Gender**			0.77
Female	94 (26%)	103 (24.9%)	
Male	267 (74%)	311 (75.1%)	
**Pack-years of tobacco use**			<0.0001
Never	62 (17.2%)	137 (33.1%)	
0 to <20	60 (16.7%)	116 (28.0%)	
> = 20	239 (66.2%)	161 (38.9%)	
**Alcohol consumption, average drinks per week**			<0.0001
<8	122 (37.9%)	239 (58.0%)	
> = 8	200 (62.1%)	173 (42.0%)	
Missing	39	2	
**HPV L1 serology Positive:Negative (%)**			
HPV16	70:291 (19%)	14:400 (3.4%)	<0.0001
HPV18	43:318 (12%)	30:384 (7.2%)	<0.04
HPV11	35:326 (9.7%)	17:397 (4.1%)	<0.003
HPV31	37:324 (10.2%)	22:392 (5.3%)	<0.01
HPV33	15:346 (4.2%)	9:405 (2.2%)	<0.17
HPV35	33:328 (9.1%)	14:400 (3.3%)	<0.001
HPV45	18:343 (5.0%)	12:402 (2.9%)	<0.19
HPV52	19:342 (5.3%)	3:411 (0.7%)	<0.0003
HPV58	20:341 (5.5%)	6:408 (1.4%)	<0.003
**Tumor sites**			
Oral	148 (41.0%)		
Pharynx	148 (41.0%)		
Larynx	65 (18.0%)		

We evaluated the association between genotype and HPV serology (Table 2); given the very low seroprevalence among controls (Table 1), it was necessary to test this association using data from all subjects. *TMC8* genotype was associated with HPV18 seropositivity (OR_AT_: 0.65, 95% CI 0.38–1.11, OR_TT_: 0.48, 95% CI 0.24–0.99), with similar non-significant trends observed for HPV16 (OR_AT_: 0.91, 95% CI 0.54–1.55, OR_TT_: 0.58, 95% CI 0.22–1.17), HPV31 (OR_AT_: 0.60, 95% CI 0.33–1.09, OR_TT_: 0.48, 95% CI 0.22–1.04), and HPV52 (OR_AT_: 0.25, 95% CI 0.09–0.67, OR_TT_: 0.36, 95% CI 0.11–1.13). We further examined the association between genotype and serology among oropharyngeal cancer cases; similar OR’s were observed, but with statistical imprecision (data not shown).

**Table 2:** Association between *TMC8* genotype and HPV L1 seropositivity—all subjects.

	*TMC8* genotype^a^				
HPV type	AA	AT	TT	OR_AT vs AA_ (95% CI)^b^	OR_TT vs AA_ (95% CI)^b^
High risk					
HPV16	25/176	45/350	14/165	0.91 (0.54–1.55)	0.58 (0.29–1.17)
HPV18	26/175	35/360	12/167	0.65 (0.38–1.11)	0.48 (0.24–0.99)
Other types					
HPV11	13/188	25/370	14/165	0.98 (0.49–1.95)	1.23 (0.56–2.69)
HPV31	22/179	27/368	10/169	0.60 (0.33–1.09)	0.48 (0.22–1.04)
HPV33	8/193	11/384	5/174	0.70 (0.28–1.76)	0.69 (0.22–2.15)
HPV35	14/187	24/371	9/170	0.87 (0.44–1.73)	0.70 (0.30–1.66)
HPV45	10/191	16/379	4/175	0.82 (0.36–1.84)	0.43 (0.13–1.41)
HPV52	12/189	6/389	4/175	0.25 (0.09–0.67)	0.36 (0.11–1.13)
HPV58	10/191	10/385	6/173	0.51 (0.21–1.24)	0.66 (0.23–1.85)

^a^ number of individuals L1 positive/L1 negative.

^b^ All OR’s adjusted for sex and age.

*TMC8* genotype was also associated with a reduced risk of all types of HNSCC (Table 3). Compared with *TMC8* AA genotype, AT and TT genotypes were inversely associated with HNSCC (OR = 0.63; 95% CI: 0.45–0.89 and OR = 0.4954; 95% CI: 0.36–0.81 respectively). These findings were consistent across anatomic sites of HNSCC (Table 3). To determine whether the genotype association was independent of HPV serology, we conducted subgroup analysis among subjects seronegative for HPV16 as well as those seronegative for all tested HPV types (Table 4). Consistent with the previous analyses *TMC8* genotype was inversely associated with HNSCC among those seronegative for HPV16 (OR_AT_ = 0.67; 95% CI: 0.45–1.03; OR_TT_ = 0.58; 95% CI: 0.34–1.00); this relationship remained for those negative for all HPV types (OR_AT_ = 0.713; 95% CI: 0.45–1.11; OR_TT_ = 0.54; 95% CI: 0.31–0.93). Subgroup analysis restricted to those seropositive was not possible given the low prevalence of seropositivity among controls (Table 1).

**Table 3:** Association between *TMC8* genotype and risk of HNSCC.

	All sites		Oral cavity		Pharynx		Larynx	
*TMC8* genotype	n^a^	OR^b^ (95% CI)	n^a^	OR^b^ (95% CI)	n^a^	OR^b^ (95% CI)	n^a^	OR^b^ (95% CI)
AA	113/142	referent	113/64	referent	113/52	referent	113/26	referent
AT	278/212	0.63	278/83	0.56	278/91	0.69	278/38	0.64
		(0.45–0.89)		(0.36–0.87)		(0.44–1.04)		(0.34–1.21)
TT	144/88	0.54	144/31	0.42	144/41	0.60	144/16	0.49
		(0.36–0.81)		(0.24–0.73)		(0.35–1.01)		(0.23–1.07)

^a^ cases/controls.

^b^ All OR’s adjusted for sex, age, packyears smoked and average drinks per week.

**Table 4:** Odds ratios for HNSCC and *TMC8* genotype among seronegative individuals.

	Seronegative for HPV16		Seronegative for all HPV types	
*TMC8* genotype	n^a^	OR^b^ (95% CI)	n^a^	OR^b^ (95% CI)
AA	83/84	referent	72/68	referent
AT	128/197	0.67 (0.43–1.03)	121/180	0.71 (0.45–1.11)
TT	49/100	0.58 (0.34–1.00)	46/94	0.54 (0.31–0.93)

^a^ cases/controls.

^b^ OR’s adjusted for sex, age, packyears smoked and average drinks per week.

## Discussion

In our case-control study of head and neck cancers, we find evidence that genetic variation in *TMC8* is inversely associated with seropositivity to high-risk HPV types. Further, *TMC8* variant genotype is inversely associated with risk of HNSCC, independent of anatomic location, and independent of HPV status.

Previously, we reported that this same genetic variation in *TMC8* was positively associated with cutaneous-type HPV infection [2]. At first glance, these strikingly different results between HPV types are surprising. However, if we consider the structural differences in the alpha and beta HPV genomes, a model emerges based on the viral E5 protein. One important difference between cutaneous (beta) HPVs and high-risk (alpha) types is presence of the E5 protein. In high-risk HPV, the E5 protein directly binds to the EVER/ZnT-1 complex [15]. As Larzarczyk explains, the EVER/ZnT-1 complex is a restriction point for HPV infection whereby alteration of this complex (and its subsequent dysregulation of zinc) is necessary for infection to occur [1]. It is plausible that the *TMC8* polymorphism alters the binding potential of the HPV E5 protein to the EVER/ZnT-1 complex, and this lessens the probability of a productive HPV infection. In contrast, the cutaneous type HPVs do not rely on E5 for productive infection (as the cutaneous HPV genome is devoid of E5). Gaud et al have demonstrated that the *TMC8* polymorphism alters the ability of EVER2 to interact with TRADD, thereby altering apoptosis potential [16]. We hypothesize that the common genetic variation in *TMC8* influences HPV susceptibility by two different mechanisms, dependent on the presence of absence of the E5 protein. *In vitro* experiments will be necessary to definitively answer these questions.

While EVER2 expression in keratinocytes has received considerable attention, given its role in the inherited syndrome Epidermodysplasia Verruciformis, recent work demonstrates that EVER2 expression in T lymphocytes exceeds that found in keratinocytes. In T-cells this high expression of EVER2 is rapidly down-regulated upon TCR stimulation, leading to an increase in intracellular Zn levels. We hypothesize that the *TMC8* polymorphism modulates this biology, impacting T-cell surveillance functions and cancer risk. Additional epidemiologic studies of non-viral mediated cancers will help elucidate whether *TMC8* is a generalized cancer susceptibility factor.

There are several limitations to the data in this study. The serologic endpoint used is highly specific for a high-risk HPV infection. However, it must be noted that serology as an endpoint has limited sensitivity, as not all individuals mount a serologic response with HPV infection. In the context of our hypothesis concerning *TMC8* genotype and susceptibility to infection, this potential limitation in sensitivity would bias our results to the null. Another limitation of these data is that the serologic response we assess is not necessarily reflective of an infection at the tumor site, but might instead reflect a high-risk infection at a site other than the tumor. However, the low seroprevalence of high-risk HPV among non-diseased individuals (Table 1) indicates this is likely not a large misclassification problem. Finally, because of this low seroprevalence of the high-risk HPV types in our control population we are unable to test whether *TMC8* genotype is associated with infection in non-diseased individuals, and this will be important to address in future work.

Of interest, a previous study of cervical cancer by Wang et al, using a GWAS approach, identified a rare SNP in the *TMC6/8* region as associated with HPV16 viral persistence (rs9893818, A allele frequency 2.5%) [5], indicating that genetic variation beyond the common SNP presented here may be important for understanding HPV infection. A recent study by Castro et al [6], reported two additional common SNPs in the *TMC6/TMC8* gene were associated with cervical cancer, however this study lacked measures on HPV (although it would be implied among cases). Clearly a comprehensive evaluation of the extent, character and phenotype of variation in the *TMC6*/*TMC8* gene region will serve to more fully delineate our understanding of genetic susceptibility to HPV infection. Finally, given our very strong associations between *TMC8* genotype and HNSCC in those without evidence of HPV infection it will be important to establish whether the *TMC8* gene is a generalized cancer susceptibility factor.
